# Assessing the capacity of Malawi’s district and central hospitals to manage traumatic diaphyseal femoral fractures in adults

**DOI:** 10.1371/journal.pone.0225254

**Published:** 2019-11-20

**Authors:** Kiran J. Agarwal-Harding, Linda Chokotho, Sven Young, Nyengo Mkandawire, Mabvuto Chawinga, Elena Losina, Jeffrey N. Katz

**Affiliations:** 1 Harvard Combined Orthopaedic Residency Program, Boston, MA, United States of America; 2 The Orthopaedic and Arthritis Center for Outcomes Research, Brigham and Women’s Hospital, Boston, MA, United States of America; 3 Department of Orthopedics, Queen Elizabeth Central Hospital, Blantyre, Malawi; 4 Department of Orthopedics, Haukeland University Hospital, Bergen, Norway; 5 Department of Orthopedics, Kamuzu Central Hospital, Lilongwe, Malawi; 6 College of Medicine, University of Malawi, Blantyre, Malawi; 7 Department of Clinical Services, Malawi Ministry of Health, Lilongwe, Malawi; 8 Department of Biostatistics, Boston University School of Public Health, Boston, MA, United States of America; 9 Departments of Epidemiology and Environmental Health, Harvard T.H. Chan School of Public Health, Boston, MA, United States of America; Technion - Israel Institute of Technology, ISRAEL

## Abstract

**Background:**

The burden of musculoskeletal trauma is growing worldwide, disproportionately affecting low-income countries like Malawi. However, resources required to manage musculoskeletal trauma remain inadequate. A detailed understanding of the current capacity of Malawian public hospitals to manage musculoskeletal trauma is unknown and necessary for effective trauma system development planning.

**Methods:**

We developed a list of infrastructure, manpower, and material resources used during treatment of adult femoral shaft fractures–a representative injury managed non-operatively and operatively in Malawi. We identified, by consensus of at least 7 out of 10 experts, which items were essential at district and central hospitals. We surveyed orthopaedic providers in person at all 25 district and 4 central hospitals in Malawi on the presence, availability, and reasons for unavailability of essential resources. We validated survey responses by performing simultaneous independent on-site assessments of 25% of the hospitals.

**Results:**

No district or central hospital in Malawi had available all the essential resources to adequately manage femoral fractures. On average, district hospitals had 71% (range 41–90%) of essential resources, with at least 15 of 25 reporting unavailability of inpatient ward nurses, x-ray, external fixators, gauze and bandages, and walking assistive devices. District hospitals offered only non-operative treatment, though 24/25 reported barriers to performing skeletal traction. Central hospitals reported an average of 76% (71–85%) of essential resources, with at least 2 of 4 hospitals reporting unavailability of full blood count, inpatient hospital beds, a procedure room, an operating room, casualty/A&E department clinicians, orthopaedic clinicians, a circulating nurse, inpatient ward nurses, electrocardiograms, x-ray, suture, and walking assistive devices. All four central hospitals reported barriers to performing skeletal traction. Operative treatment of femur fracture with a reliable supply of implants was available at 3/4 hospitals, though 2/3 were dependent entirely on foreign donations.

**Conclusion:**

We identified critical deficiencies in infrastructure, manpower, and essential resources at district and central hospitals in Malawi. Our findings provide evidence-based guidance on how to improve the musculoskeletal trauma system in Malawi, by identifying where and why essential resources were unavailable when needed.

## Introduction

Trauma accounts for an increasing burden of death and disability worldwide, disproportionately affecting low- and middle-income countries (LMICs) [1–4]. Short- and long-term trauma-related disability can be especially crippling for the poorest patients who can enter a vicious cycle of poverty due to associated healthcare costs and decreased productivity [5, 6]. Due in large part to a rising number of road injuries, musculoskeletal impairment is increasingly common in LMICs [2, 3, 7–12]. Significant injury-related disability is preventable with quality trauma and surgical care, which unfortunately remains out of reach for many patients, especially in LMICs [8, 13–18].

Malawi is a low-income country in southeastern Africa of about 19 million people, 83% of whom live in rural areas [19, 20]. Traumatic injuries and musculoskeletal impairment are common, though the resources to manage musculoskeletal trauma remain inadequate [12, 21–26]. About 60% of total healthcare services in Malawi are provided by public hospitals, which are organized into three levels [27]. The primary level is a health center, providing basic medical and maternity care, and no surgical care. The secondary level is a district hospital staffed by general doctors and clinical officers, providing non-specialized surgical care. The tertiary level is a central hospital, which provides specialist care. Trauma care is offered at district and central hospitals, though the availability of essential resources to safely manage patients with musculoskeletal injuries is largely unknown [28, 29].

Diaphyseal femoral fracture is a common injury treated in Malawian hospitals [30]. We have previously found that every week in Malawi, approximately one adult patient with femoral shaft fracture presents to each district hospital and 4 present to each central hospital [31, 32]. They are largely treated non-operatively with skeletal traction, with operative treatment available only at the central hospitals [33]. For additional details regarding musculoskeletal trauma care and the burden of femoral shaft fracture in Malawi, see the supplementary appendix (S1 File). We sought to assess the musculoskeletal trauma care capacity of all public district and central hospitals in Malawi, under the jurisdiction of the Ministry of Health, by examining capacity in the context of non-operative and operative treatment of femoral shaft fracture as a representative injury.

## Methods

### Capacity survey tool

We developed a capacity assessment survey examining femoral shaft fracture management (S1 File). We adapted the methodology performed by Stewart et al in 2015 during their strategic assessment of trauma care capacity in Ghana [34]. We first identified items relevant to musculoskeletal trauma in the WHO Guidelines for Essential Trauma Care (GETC) [35], and used our own clinical experience in Malawi to create a list of 51 important items of infrastructure, manpower, and material resources (Table 1). We assessed three phases of care: 1) initial management, including resuscitation, treatment of open fracture, stabilization/immobilization, and admission; 2) definitive treatment, including non-operative and operative treatment; and 3) aftercare, including pain management, discharge planning, mobilization, and rehabilitation. In each hospital, we assessed whether each item was routinely present, and each item’s actual availability in the last 7 days. We solicited reasons for unavailability of infrastructure, key procedures, and material resources. Reasons for unavailability of staff were not solicited.

**Table 1:** Infrastructure, manpower, and material resources for management of adult femoral shaft fractures in Malawi.

	Phase of care	Item availability N (%) of hospitals	
		District Hospitals	Central Hospitals
***Infrastructure***			
1. Basic chemistry panel/urea and electrolytes	IM	11 (44)	1 (25)
2. Full blood count	IM	15 (60)*	1 (25)*
3. Casualty area/accident and emergency department	IM	15 (60)	4 (100)*
4. Ambulance for patient transfer	IM	22 (88)	4 (100)
5. Inpatient hospital beds	IM/DT	20 (80)*	1 (25)*
6. Procedure room	DT	20 (80)	2 (50)*
7. Operating room	DT	19 (76)*	2 (50)*
8. Post-Anesthesia recovery room or equivalent)	DT	6 (26)	3 (75)*
9. Rehabilitation and physical therapy space	A	10 (40)	4 (100)*
***Manpower***			
10. Triage nurse (or equivalent)	IM	5 (20)	2 (50)
11. Casualty/A&E department clinicians	IM	10 (40)	2 (50)*
12. Radiology technician	IM	24 (96)*	3 (75)*
13. Orthopaedic clinical officer (OCO)	DT	22 (88)*	2 (50)*
14. Orthopaedic surgeon	DT	0 (0)	2 (50)*
15. Scrub nurse	DT	21 (84)*	4 (100)*
16. Circulating nurse	DT	17 (68)	2 (50)*
17. Inpatient ward nurses	DT	9 (36)*	2 (50)*
18. Anesthesia clinical officer (ACO)	DT	24 (96)*	4 (100)*
19. Anesthesiologist	DT	0 (0)	2 (50)
20. Discharge planning	A	8 (32)	0 (0)
21. Physiotherapist/ physiotherapy technician	A	16 (64)	4 (100)*
***Material Resources***			
22. Pulse oximeter	IM	20 (80)*	3 (75)*
23. Electrocardiograms	IM	4 (17)	2 (50)*
24. Thermometer	IM	22 (88)*	4 (100)*
25. Blood pressure cuff	IM	19 (76)*	3 (75)*
26. X-ray	IM	8 (32)*	2 (50)*
27. C-arm	DT	1 (4)	3 (75)*
28. CT	IM	0 (0)	1 (25)
29. Ultrasound	A	18 (75)	4 (100)
30. Morphine (or equivalent)	IM/DT/A	15 (60)*	4 (100)*
31. Ibuprofen (or equivalent)	IM/DT/A	15 (60)*	4 (100)*
32. Paracetamol/acetaminophen	IM/DT/A	15 (60)*	4 (100)*
33. Intravenous infusion sets (lines and cannulas)	IM/DT	24 (96)*	4 (100)*
34. Crystalloids	IM/DT	24 (96)*	4 (100)*
35. Colloids	IM/DT	7 (28)	0 (0)
36. Blood products	IM/DT	15 (60)*	3 (75)*
37. General anesthetic drugs	DT	23 (92)*	4 (100)*
38. Oral antibiotics	A	19 (76)*	4 (100)*
39. Intravenous antibiotics	IM/DT	19 (76)*	4 (100)*
40. Acetylsalicylic acid (aspirin)	A	21 (84)	2 (50)
41. Low molecular weight heparin (or equivalent)	A	0 (0)	0 (0)
42. Skin traction	DT	20 (80)*	4 (100)*
43. Skeletal traction	DT	15 (60)*	3 (75)*
44. Plaster of Paris	DT	20 (80)*	4 (100)*
45. External fixator	IM/DT	10 (40)*	4 (100)*
46. Intramedullary nail	DT	0 (0)	3 (75)*
47. Large fragment screws / plates	DT	0 (0)	3 (75)*
48. Suture	DT	21 (84)*	2 (50)*
49. Cotton wool	DT	20 (80)*	3 (75)*
50. Gauze and bandages	DT/A	10 (40)*	3 (75)*
51. Walkers, canes, crutches	A	8 (33)*	2 (50)*

Each item is assigned a dominant phase of care: indicated as Initial Management (IM), Definitive Treatment (DT), and Aftercare (A). Some items that are more broadly applicable are assigned multiple phases of care. Classification of items as essential for management of traumatic diaphyseal femoral fractures in adults was determined by modified Delphi methodology with input from study investigators and health practitioners in Malawi. Essential status is indicated at the district and central hospital level by an asterisk (*). Item availability is listed at the district and central hospital level as the number (and percentage) of hospitals where the item was present and available in the 7 days before survey completion.

### Definition of essential resources

We assembled an expert panel of 10 clinicians with experience managing femur fracture in Malawi or a similar low-resource environment. Five panelists were practicing in Malawi, two in the UK, two in the US, and one in South Africa. Eight panelists were orthopaedic surgeons, and two were orthopaedic surgery residents. We sent each expert an electronic survey with the 51 items identified by study investigators as important in the management of femoral shaft fracture. Following the same format as the WHO GETC, panelists classified items as essential, desirable, possibly required, or irrelevant at the district and central hospital level. All items marked essential by at least 7 of the 10 panelists–representing a two-thirds majority consensus [36, 37]–were considered essential (i.e. a minimum requirement) for safe management of adult femoral shaft fractures.

### Capacity survey administration

**Fig 1:**
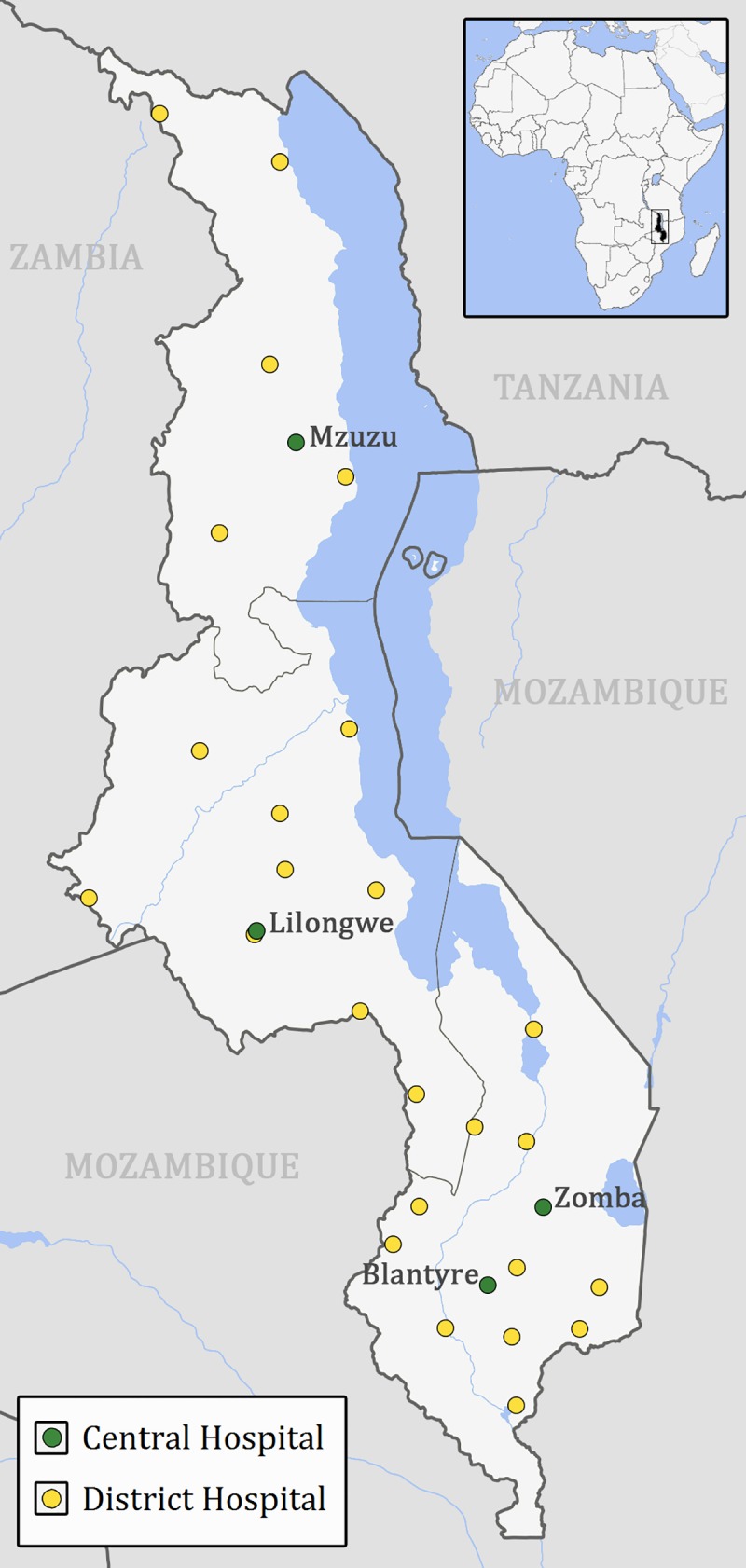
Location of all government district and central hospitals in Malawi. District hospitals are each represented by a yellow dot, central hospitals by a green dot. All hospitals are listed in S1 Table. Original maps were sourced from USGS National Map Viewer (http://viewer.nationalmap.gov/viewer/) and Maps at the CIA (https://www.cia.gov/library/publications/the-world-factbook/index.html). Figure is similar but not identical to the original images; for illustrative purposes only.

We collected capacity survey data from all 25 district and 4 central hospitals in Malawi (Fig 1, S1 Table). Between May 29 and June 15, 2018, capacity surveys were hand-delivered to each hospital’s orthopaedics department, and the first author (K.J.A.H.) performed in-person informed consent in writing of all survey respondents. An orthopaedic clinical officer (OCO)–a non-physician clinician–or an orthopaedic surgeon at each hospital completed the survey alone. To assess the validity of survey responses, we randomly selected 25% of the hospitals (6 district hospitals and 1 central hospital) by computer algorithm for additional “spot checks”. During these “spot checks”, the first author independently completed the capacity survey, personally inspecting the hospital facilities and resources, and speaking directly with hospital personnel in relevant clinical departments. “Spot checks” were performed simultaneously with the first author’s visit to each hospital, and with the assistance of staff members who had not completed the survey themselves. To minimize interruption of clinical workflow, we notified hospitals prior to the visit and if they had been selected for a “spot check”.

### Data analysis

Comparing survey respondents to the study investigators during “spot checks”, we determined inter-rater reliability by percent agreement and Cohen’s kappa statistics [38, 39]. We then examined the total number of district and central hospitals where each surveyed item was present and available in the last 7 days. For items where hospitals reported reasons for unavailability, we determined the most common reasons. Using the list of items identified as essential by our expert panel, we calculated the percentage of essential items present and available at each hospital. We examined average percentage of essential item availability by hospital level and geographic region, item type (infrastructure, manpower, material resource), and phase of care. Separately for district and central hospitals, we calculated the number of hospitals at which each item was unavailable and identified the most commonly missing essential items.

We performed all analyses using Microsoft Excel (Seattle, WA, USA) and SAS 9.4 (SAS Institute Inc., Cary, NC, USA). The College of Medicine Research Ethics Committee (COMREC P.02/18/2360) in Malawi and the Institutional Review Board at Brigham and Women’s Hospital, Boston, MA, USA provided ethical approval of the study.

## Results

### Capacity survey completion and reliability

The survey was completed by every government-run public district and central hospital in Malawi. Twenty-three district hospitals (92%) and all four central hospitals (100%) responded regarding the presence, availability, and reason for unavailability of each of the 51 surveyed items. Two district hospitals did not respond regarding post-anesthesia recovery room and low molecular weight heparin. One district hospital did not respond regarding walking assistive devices. Percent agreement of responses between survey respondents and study investigators during “spot checks” for all hospitals was 94% (range 85–99%), and Cohen’s kappa coefficient of inter-rater reliability was 0.88 (95% CI 0.84–0.91) showing substantial to near-perfect agreement (Table 2) [39].

**Table 2:** Capacity survey inter-rater reliability.

	*Percent agreement*	*Cohen's kappa* *(95% CI)*
Bwaila District Hospital	87%	0.72 (0.58–0.86)
Dowa District Hospital	96%	0.92 (0.85–1.00)
Kasungu District Hospital	98%	0.96 (0.91–1.00)
Mwanza District Hospital	94%	0.88 (0.79–0.97)
Rumphi District Hospital	97%	0.94 (0.88–1.00)
Salima District Hospital	99%	0.98 (0.94–1.00)
All District Hospitals	95%	0.91 (0.87–0.94)
Queen Elizabeth Central Hospital	85%	0.69 (0.55–0.83)
All Hospitals	94%	0.88 (0.84–0.91)

The validity of responses to the study survey was assessed by the study investigators performing “spot checks” of a randomly selected sample of 25% of the hospitals: 6 district hospitals and 1 central hospital. Percent agreement and Cohen’s kappa coefficient for inter-rater reliability are reported for each hospital, comparing survey responses from hospital staff to responses by the study investigators during “spot checks”.

### Essential resources

Essential items that would be a minimum requirement to safely manage adult femoral shaft fractures were identified by expert panel consensus. Essential items included 3 infrastructure, 5 manpower, and 21 material resources at the district hospital level; and 7 infrastructure, 9 manpower, and 25 material resources at the central hospital level (Table 1). At both the district and central hospital levels, essential items pertained primarily to monitoring of vital signs, fluid resuscitation, pain management, x-ray diagnosis of fracture, temporary fracture stabilization, non-operative fracture treatment, inpatient hospitalization, and rehabilitation. At the central hospital level, essential items additionally pertained to pre-operative evaluation and operative treatment of femoral shaft fractures.

### Essential resource availability in district hospitals

Availability of each surveyed item is presented in Table 1. No district hospitals had all essential items to safely manage femoral shaft fractures. On average, district hospitals had 71% (range 41–90%) of all essential items, 72% (range 0–100%) of essential infrastructure, 80% (range 40–100%) of essential manpower, and 69% (range 29–90%) of essential material resources. A mean of 80% (range 66–86%) of essential items were available at district hospitals in the northern region, 69% (range 41–90%) in the central region, and 69% (range 52–86%) in the southern region.

**Fig 2:**
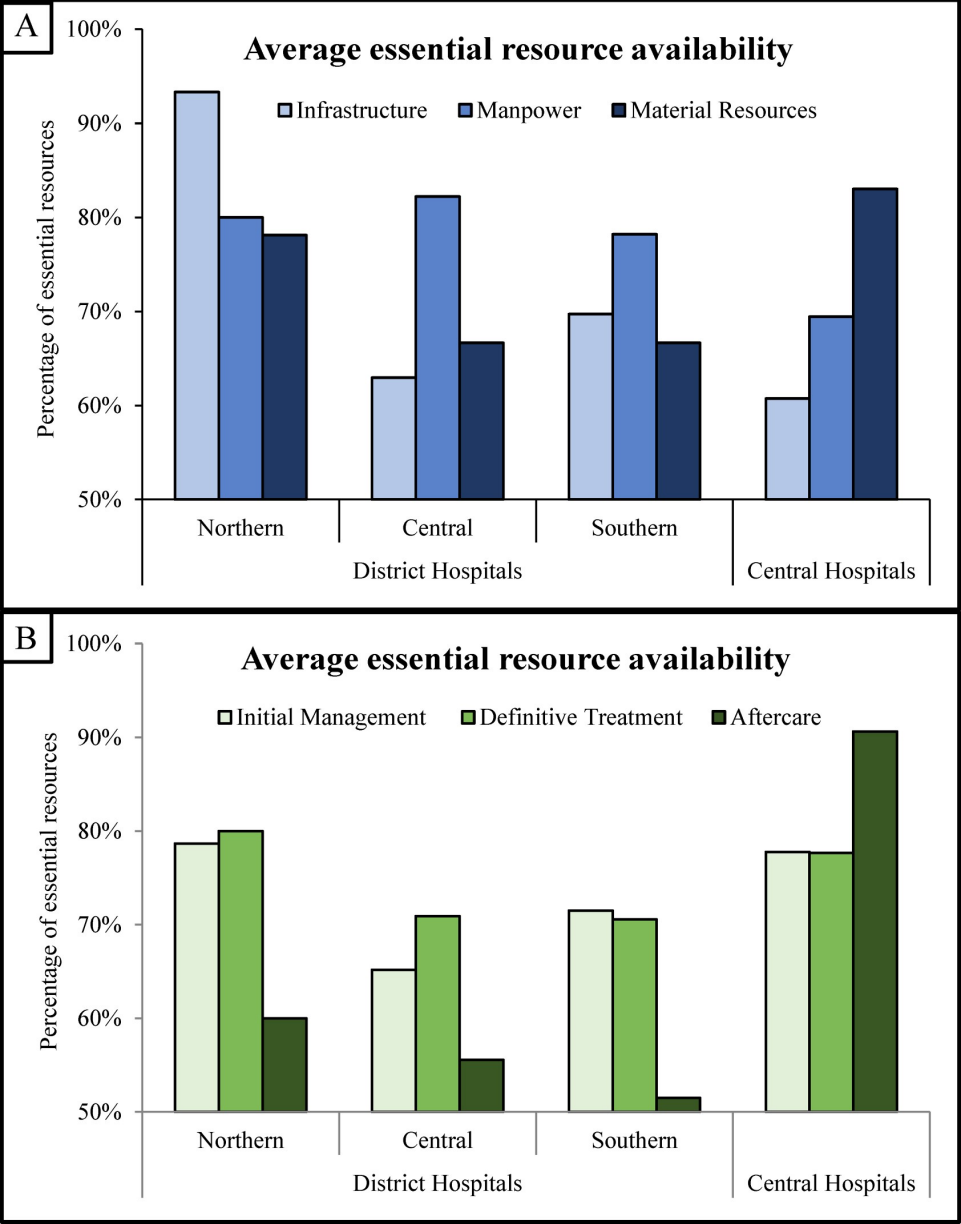
Average percentage of essential resources available in district and central hospitals. District hospitals are plotted by geographic region. Results are plotted by resource type (A) and by resource phase of care (B).

Nine district hospitals (36%) had all essential infrastructure, including 4/5 district hospitals in the northern region, 2/9 in the central region, and 3/11 in the southern region. Northern region district hospitals had the highest average availability of essential infrastructure (93%, range 67–100%), followed by the southern region (70%, range 33–100%), then the central region (63%, range 0–100%). Six district hospitals (24%) reported availability of all essential manpower, including 1/5 in the northern region, 3/9 in the central region, and 2/11 in the southern region. District hospitals in all three geographic regions, had a mean of 80% of essential manpower (northern: 80%, range 60–100%; central: 82%, range 60–100%; southern: 78%, range 40–100%). No district hospitals had all essential material resources. Northern region district hospitals had a mean of 78% (range 67–86%) of essential material resources, compared to 67% (range 29–90%) in the central region, and 67% (range 38–90%) in the southern region (Fig 2A).

District hospitals had an average of 71% (range 40–93%) of essential items for initial management and stabilization of injured patients, 73% (range 43–95%) of essential items for definite fracture treatment, and 55% (range 0–100%) of essential items for aftercare and rehabilitation. Northern region district hospitals had the highest mean availability of essential items for initial management of femoral shaft fractures (79%, range 53–93%), followed by the southern region (72%, range 53–93%), then the central region (65%, range 40–93%). Average availability of essential items for definitive treatment was 80% (range 62–90%) in the northern region, 71% (range 43–95%) in the central region, and 71% (range 48–95%) in the southern region. For aftercare and rehabilitation, northern region district hospitals had the highest average essential item availability (60%, range 33–83%), followed by the central region (56%, range 0–100%), then the southern region (52%, range 0–83%) (Fig 2B).

The following essential resources were unavailable in 10 or more of the district hospitals: full blood count, inpatient ward nurses, x-ray, pain medications, blood products, skeletal traction, external fixators, gauze and bandages, and walking assistive devices (Table 3). Almost all district hospitals (24, 96%) reported barriers to providing skeletal traction. Drills were unavailable, broken, or absent (9/24 district hospitals). OCOs reported using a mallet (2/24) or T-handle (2/24) to insert pins. Only hand drills were available (9/24), and they would jam (3/24), need grease (1/24), or would be rusted and unusable (1/24). No T-handle was available for pin insertion/removal (5/24). There were inadequate supplies of weights (11/24), forcing providers to improvise solutions like using stones or bricks (3/24). There were inadequate supplies of appropriate hospital beds, functioning traction frames, or pulleys (15/24); inadequate supplies of traction pins (12/24), with pins being reused and becoming dull (1/24), and plaster being used to attach weights to pins (1/24). Staff members were reportedly unavailable at night or on the weekend (3/24), were inadequately trained to perform skeletal traction (1/24) and were inadequately trained to care for patients in skeletal traction (2/24).

**Table 3:** Unavailable essential resources in Malawian district hospitals (n = 25).

	Items unavailable in…		
	>50% of hospitals	26–50% of hospitals	10–25% of hospitals
**Infrastructure**		• Full blood count (10)	• Operating room (6) • Inpatient hospital beds (5)
**Manpower**	• Inpatient ward nurses (16)		• Scrub nurse (4) • Orthopaedic clinical officer (3)
**Material Resources**	• X-ray (17) • Walkers, canes, crutches (17) • External fixator (15) • Gauze and bandages (15)	• Morphine, ibuprofen, paracetamol/acetaminophen (10) • Blood products (10) • Skeletal traction (10)	• Blood pressure cuff (6) • Oral and IV antibiotics (6) • Pulse oximeter (5) • Skin traction (5) • Plaster of Paris (5) • Cotton wool (5) • Suture (4) • Thermometer (3)

Items missing from >50%, 26–50%, and 10–25% of district hospitals are listed by item category (infrastructure, manpower, and material resources). The number of district hospitals where each item was missing or unavailable is included in parenthesis.

At the time of survey completion, skin traction–a temporary measure using strapping/adhesive tape applied to the skin for traction–was unavailable within the last 7 days in 5 (20%) of the district hospitals, with 19 hospitals (76%) reporting barriers to providing this treatment. Barriers included inadequate supplies of tape (10/19 district hospitals), with one hospital reporting that patients often had to buy their own; poor quality of tape (10/19), with the zinc oxide tape reportedly causing rashes/blisters (2/19). Staff members were reportedly inadequately trained to care for patients in skin traction (3/19) and were also unavailable at night or on the weekend (2/19).

### Essential resource availability in central hospitals

No central hospitals had all essential items to safely manage femoral shaft fractures. Central hospitals had an average of 76% (range 71–85%) of all essential items—61% (range 43–71%) of essential infrastructure, 69% (range 56–78%) of essential manpower, and 83% (range 76–92%) of essential material resources (Fig 2A). The average central hospital had 78% (range 72–83%) of essential items for initial management, 78% (range 71–93%) for definitive treatment, and 91% (range 88–100%) for aftercare (Fig 2B).

The following items were absent or unavailable from two or more of the four hospitals: full blood count, inpatient hospital beds, procedure room, operating room, casualty/accident and emergency (A&E) department clinicians, orthopaedic clinical officers, orthopaedic surgeon, circulating nurse, inpatient ward nurses, electrocardiograms, x-ray, suture, and walking assistive devices (Table 4). Though unavailable in the last 7 days at only one central hospital, all central hospitals reported barriers to providing skeletal traction. Drills were unavailable or absent (2/4 central hospitals); there was inadequate supply of traction pins (1/4), weights (1/4), and traction frames (2/4); and staff members were reportedly inadequately trained to perform skeletal traction or care for patients in skeletal traction (1/4).

**Table 4:** Unavailable essential resources in Malawian central hospitals (n = 4).

	Items unavailable in…		
	Three hospitals	Two hospitals	One hospital
**Infrastructure**	• Full blood count (3) • Inpatient hospital beds (3)	• Procedure room (2) • Operating room (2)	• Post-Anesthesia recovery room or equivalent (1)
**Manpower**		• Casualty/A&E department clinicians (2) • Orthopaedic clinical officer (2) • Orthopaedic surgeon (2) • Circulating nurse (2) • Inpatient ward nurses (2)	• Radiology technician (1)
**Material Resources**		• Electrocardiograms (2) • X-ray (2) • Suture (2) • Walkers, canes, crutches (2)	• Pulse oximeter (1) • Blood pressure cuff (1) • C-arm (1) • Blood products (1) • Skeletal traction (1) • Intramedullary nail (1) • Large fragment screws / plates (1) • Cotton wool (1) • Gauze and bandages (1)

Items missing from three, two, and one of the central hospitals are listed by item category (infrastructure, manpower, and material resources). The number of central hospitals where each item was missing or unavailable is included in parenthesis.

Operative management of femoral shaft fractures was available at 3 central hospitals; unavailable at one while the orthopaedic surgeon was on holiday at the time of survey completion. In all 3 central hospitals where operative treatment was available, IM nails and large fragment sets were present, available, and with reliable supply. Two hospitals reported relying entirely on donations by visiting surgeons.

## Discussion

We found that no district or central hospital in Malawi had available the minimum required essential resources to safely manage adult femoral shaft fractures. On average, only 71% of these essential resources were available in district hospitals, and 76% in the central hospitals. Though non-operative treatment remains the first-line treatment in Malawi, 96% of district hospitals and all 4 central hospitals reported barriers to performing skeletal traction. Operative treatment with a reliable supply of implants was available at 3 of the 4 central hospitals, though most central hospitals were entirely dependent on foreign donations, further limiting availability of surgical care.

The WHO GETC state that, at the district hospital level, basic immobilization is essential, skin and skeletal traction are possibly required, closed reduction of fractures is possibly required, and open reduction of fractures is irrelevant [35]. Our expert panel felt that resources necessary for immobilization, skin and skeletal traction, and closed reduction of fractures were all essential for Malawian district hospitals. This seems reasonable as Malawian district hospitals manage most of the musculoskeletal trauma nationwide [40, 41]. Our expert panel agreed with the WHO GETC that all of these services are essential at tertiary care (central) hospitals [35].

In a web-based survey of 267 district and central hospitals throughout East, Central, and Southern Africa by Chokotho et al, only 31% reported formal emergency departments to manage traumatic injuries [26]. In Malawi, we found that 60% of district hospitals and 100% of central hospitals reported availability of a casualty/A&E department, although we did not assess their actual functionality or capacity to manage trauma patients in depth. Chokotho et al reported that C-arms and CT scanner were available in fewer than 5% of district hospitals and in about 25% of central hospitals in the region [26]. We similarly found that only one district hospital in Malawi (4%) reported having a working C-arm, no district hospitals reported CT scanners, and only one central hospital (25%) had a functioning CT. We found that 2/4 of central hospitals had working c-arms that produced adequate quality images. Chokotho et al reported that closed fracture care was available in 72% of surveyed hospitals, and the tools necessary to surgically treat fractures were available in only 20% of district and 49% of central hospitals [26]. We similarly found that skeletal traction to non-operatively treat adult femoral shaft fractures was available in 15/25 (60%) of district hospitals and 3/4 of central hospitals. However, almost all hospitals reported barriers to providing this treatment, with many reporting inadequate or absent drills, and insufficient supply of traction pins, weights, and traction frames. IM nails and large fragment sets for operative treatment were present only at the central hospitals, and available when needed in 3/4 of central hospitals. However, at the time of this survey only 32% of district and 50% of central hospitals had adequate availability of x-ray to diagnose fractures, which our expert panel regarded as essential in both non-operative and operative treatment of fracture. No district or central hospital in Malawi had available all the essential resources to adequately manage adult femoral shaft fractures, though one district hospital had 90% and one central hospital had 85%. Ten percent of central hospitals in the study by Chokotho et al reported a sustainable supply of implants [26]. We observed higher rates of reliable implant supply among Malawian central hospitals, though with a complete dependency on foreign donations.

Malawi, like many other sub-Saharan African nations, is rapidly urbanizing [19]. With a growing urban population, rising trauma burden due to road traffic injuries, and centralization of surgical care at central hospitals, there will be a growing demand on urban central hospitals to provide essential operative musculoskeletal trauma care. At the time of survey completion, central hospitals in Malawi lacked essential capacity, most notably having insufficient infrastructure and manpower. Only one central hospital reported having beds available for newly admitted patients with musculoskeletal injuries, while the other three central hospitals reported that there were more patients than beds available. In contrast, 80% of district hospitals had beds available for musculoskeletal trauma patients. This apparent “excess” bed capacity is in line with earlier surveys of Malawian district hospitals [42]. However, inpatient ward nurses were in short supply throughout Malawi, and hospitals reported that ward nurses were inadequately trained to care for patients in skin or skeletal traction. Infrastructure development and advanced skills training must be prioritized to improve the trauma system.

As of November 2018, with the assistance of external donor funding, construction began on the Lilongwe Institute of Orthopaedics and Neurosurgery (LION), which should greatly expand operative infrastructure at Kamuzu Central Hospital, and for the country as a whole [43]. With developments of this kind, patients presenting to district hospitals with injuries needing surgery could be transferred to a central hospital with a “short stay trauma service” for treatment, then returned to the district hospitals for surgical aftercare and rehabilitation, allowing faster scale up of services and better utilization of capacity at the district level. However, our study demonstrates that health system strengthening must occur simultaneously in the district and central hospitals, with interventions appropriate to the level of service delivery. We found that ambulances were widely available for patient transfer between the district and central hospitals. However, essential resources for aftercare and rehabilitation of femoral fracture patients were notably deficient in district hospitals. A rehabilitation and physical therapy space was available in only 40% of districts, most reporting insufficient stock of necessary equipment and/or an inadequate amount of dedicated space. Walking assistive devices were available in only 33% of hospitals, with hospitals relying on donations from a local NGO or having patients commission their own from private carpenters. Pain medications were available in only 60% of district hospitals. Gauze and bandages were available in only 40% of district hospitals, 80% of which reported a short supply.

With few specialist orthopaedic surgeons currently available in Malawi, centralization of operative capabilities remains critical to scale up surgical services, train further specialists, and serve the district hospitals more efficiently. However, the district hospitals will continue to play an important role in delivering trauma care for the majority rural population. Although operative treatment is the international gold standard for diaphyseal femoral fractures [44], non-operative treatment remains the standard of care in Malawi, and the only treatment available in district hospitals. There is an urgent need in the short term to improve the capacity to perform skeletal traction especially in district hospitals, otherwise the provision of a suboptimal treatment in the context of inadequate resources may result in catastrophic patient outcomes. In the intermediate term, centralized short stay trauma services can provide scaled up operative treatment also to the districts. Eventually, once enough trained specialists are available in central hospitals to sustainably run the clinical and training programs, operative capacity will need to be decentralized to the district hospitals as well, to meet the needs of a modernizing Malawi.

This study has several limitations. First, relying on a single individual at a hospital to provide an overview of adult femur fracture care may have provided a limited impression of a hospital’s capacity. This may be especially true at central hospitals, where larger teams of providers share responsibility in patient care. However, we found high inter-rater reliability between study participants and the study investigators during independent “spot-checks”, including at the central hospital. Second, as with many survey methodologies, there is potential for social desirability bias. Respondents may have felt pressured to either over-report capacity for fear of retaliation from their superiors, or under-report capacity under the assumption that the study investigators desired these kinds of responses. Respondents may have over-reported missing or unavailable items with the hope that this would expedite resource replenishment. All surveys were completed anonymously to protect participants’ identities, though the hospital where each respondent worked was recorded. The high reliability of survey respondents compared to the independent “spot checks” likely indicates that this bias played a minimal role in this study. Third, we used femoral shaft fracture as a representative injury to assess musculoskeletal trauma care capacity. As femur fractures in Malawi are generally admitted from the outpatient or A&E department to the inpatient ward, and then treated either non-operatively or operatively, we felt focusing on this injury allowed for assessment of resources throughout the orthopaedic departments and for all phases of care. While an injury like ankle fracture may better elucidate resource limitations for fractures that are routinely treated in the outpatient setting, our methodology still allowed for assessment of x-ray, plaster of Paris, pain medication, walking assistive devices, and gauze and bandages, all of which are broadly relevant. Fourth, we collected data during a three-week period in the dry season. Fluctuations in disease burden in Malawi have been reported [45], and may be associated with seasonal variations in resource availability. Though the reason remains unclear, fewer patients with acute fractures present to orthopaedic clinics during the dry season [46]. We may have therefore overestimated capacity, compared to during the rainy season when higher trauma burden may result in greater resource utilization. A repeat assessment of trauma capacity is warranted in the rainy season, and continuous monitoring of resource availability should be a goal for the future, to allow for better supply chain management and real-time adaptation to the country’s needs.

Using femoral shaft fracture as a representative injury, we have evaluated the musculoskeletal trauma care capacity of every district and central hospital in Malawi, and found significant deficiencies in infrastructure, manpower, and material resources. Our findings provide evidence to help guide the development of the musculoskeletal trauma system in Malawi to meet the needs of a growing population with an increasing burden of injuries. Evidence-based policy planning could take into consideration the unavailability of essential resources, as well as the specific reasons for unavailability, which we have gathered from each hospital in this study. The cost and feasibility of addressing each specific item’s availability should be examined in future studies. Strategic planning for trauma and orthopaedic system development in Malawi should take into consideration the role of centralizing and decentralizing certain capabilities, and preparing the system to adapt to future challenges.

## Supporting information

S1 File Supplemental appendix. Includes additional study context regarding musculoskeletal trauma care and femoral shaft fractures in Malawi, and the capacity assessment survey tool.
(DOCX)

S1 Table List of government district and central hospitals in Malawi. (DOCX)

## References

[1] Lozano R, Naghavi M, Foreman K, Lim S, Shibuya K, Aboyans V, et al Global and regional mortality from 235 causes of death for 20 age groups in 1990 and 2010: a systematic analysis for the Global Burden of Disease Study 2010. Lancet (London, England). 2012;380(9859):2095–128. 10.1016/S0140-6736(12)61728-0 PMC10790329 23245604

[2] Vos T, Flaxman AD, Naghavi M, Lozano R, Michaud C, Ezzati M, et al Years lived with disability (YLDs) for 1160 sequelae of 289 diseases and injuries 1990–2010: a systematic analysis for the Global Burden of Disease Study 2010. Lancet (London, England). 2012;380(9859):2163–96. 10.1016/S0140-6736(12)61729-2 23245607 PMC6350784

[3] Peden M, Scurfield R, Sleet D, Mohan D, Hyder A, Jarawan E, et al World Report on Road Traffic Injury Prevention. Geneva, Switzerland: World Health Organization; 2004.

[4] Kotagal M, Agarwal-Harding KJ, Mock C, Quansah R, Arreola-Risa C, Meara JG. Health and economic benefits of improved injury prevention and trauma care worldwide. PloS One. 2014;9(3):e91862 10.1371/journal.pone.0091862 24626472 PMC3953529

[5] Gosselin RA, Spiegel DA, Coughlin R, Zirkle LG. Injuries: the neglected burden in developing countries. Bulletin of the World Health Organization. 2009;87(4):246a. S0042-96862009000400002 [pii]. 19551225 10.2471/BLT.08.052290 PMC2672580

[6] Global status report on road safety 2013: supporting a decade of action. Geneva, Switzerland: World Health Organization; 2013.

[7] Murray CJ, Vos T, Lozano R, Naghavi M, Flaxman AD, Michaud C, et al Disability-adjusted life years (DALYs) for 291 diseases and injuries in 21 regions, 1990–2010: a systematic analysis for the Global Burden of Disease Study 2010. Lancet (London, England). 2012;380(9859):2197–223. 10.1016/S0140-6736(12)61689-4 23245608

[8] Mock C, Cherian MN. The global burden of musculoskeletal injuries: challenges and solutions. Clinical Orthopaedics and Related Research. 2008;466(10):2306–16. 10.1007/s11999-008-0416-z 18679760 PMC2584305

[9] Spiegel DA, Gosselin RA, Coughlin RR, Joshipura M, Browner BD, Dormans JP. The burden of musculoskeletal injury in low and middle-income countries: challenges and opportunities. The Journal of Bone and Joint Surgery American volume. 2008;90(4):915–23. 10.2106/JBJS.G.00637 18381331

[10] Matheson JI, Atijosan O, Kuper H, Rischewski D, Simms V, Lavy C. Musculoskeletal impairment of traumatic etiology in Rwanda: prevalence, causes, and service implications. World Journal of Surgery. 2011;35(12):2635–42. 10.1007/s00268-011-1293-2 21964816

[11] Elliott IS, Groen RS, Kamara TB, Ertl A, Cassidy LD, Kushner AL, et al The burden of musculoskeletal disease in Sierra Leone. Clinical Orthopaedics and Related Research. 2015;473(1):380–9. 10.1007/s11999-014-4017-8 25344406 PMC4390972

[12] Varela C, Young S, Groen R, Banza L, Mkandawire NC, Viste A. Untreated surgical conditions in Malawi: A randomised cross-sectional nationwide household survey. Malawi Medical Journal. 2017;29(3):231–6. Epub 2018/06/07. 10.4314/mmj.v29i3.1 29872512 PMC5811994

[13] Alkire BC, Raykar NP, Shrime MG, Weiser TG, Bickler SW, Rose JA, et al Global access to surgical care: a modelling study. The Lancet Global Health. 2015;3(6):316 10.1016/S2214-109X(15)70115-4 25926087 PMC4820251

[14] Spiegel DA, Nduaguba A, Cherian MN, Monono M, Kelley ET. Deficiencies in the availability of essential musculoskeletal surgical services at 883 health facilities in 24 low- and lower-middle-income countries. World Journal of Surgery. 2015;39(6):1421–32. 10.1007/s00268-015-2971-2 25663008

[15] Shrime MG, Daniels KM, Meara JG. Half a billion surgical cases: Aligning surgical delivery with best-performing health systems. Surgery. 2015;158(1):27–32. 10.1016/j.surg.2015.03.025 25934078 PMC4461452

[16] Funk LM, Weiser TG, Berry WR, Lipsitz SR, Merry AF, Enright AC, et al Global operating theatre distribution and pulse oximetry supply: an estimation from reported data. Lancet (London, England). 2010;376(9746):1055–61. 10.1016/S0140-6736(10)60392-3 20598365

[17] Meara JG, Leather AJ, Hagander L, Alkire BC, Alonso N, Ameh EA, et al Global Surgery 2030: evidence and solutions for achieving health, welfare, and economic development. Lancet (London, England). 2015;386(9993):569–624. 10.1016/S0140-6736(15)60160-X 25924834

[18] Linden AF, Sekidde FS, Galukande M, Knowlton LM, Chackungal S, McQueen KA. Challenges of surgery in developing countries: a survey of surgical and anesthesia capacity in Uganda's public hospitals. World Journal of Surgery. 2012;36(5):1056–65. 10.1007/s00268-012-1482-7 22402968

[19] Health Nutrition and Population Statistics [Internet]. The World Bank. 2017 [cited March 20, 2019]. Available from: https://datacatalog.worldbank.org/dataset/health-nutrition-and-population-statistics.

[20] World Development Indicators [Internet]. The World Bank. 2017 [cited March 20, 2019]. Available from: https://datacatalog.worldbank.org/dataset/world-development-indicators.

[21] Young S, Banza L, Munthali BS, Manda KG, Gallaher J, Charles A. The impact of the increasing burden of trauma in Malawi on orthopedic trauma service priorities at Kamuzu Central Hospital. Acta Orthopaedica. 2016;87(6):632–6. 10.1080/17453674.2016.1228413 27587339 PMC5119448

[22] Kiser MM, Samuel JC, McLean SE, Muyco AP, Cairns BA, Charles AG. Epidemiology of pediatric injury in Malawi: burden of disease and implications for prevention. International Journal of Surgery (London, England). 2012;10(10):611–7. 10.1016/j.ijsu.2012.10.004 23142508

[23] Samuel JC, Akinkuotu A, Villaveces A, Charles AG, Lee CN, Hoffman IF, et al Epidemiology of injuries at a tertiary care center in Malawi. World Journal of Surgery. 2009;33(9):1836–41. 10.1007/s00268-009-0113-4 19597877 PMC3290404

[24] Chokotho L, Mulwafu W, Jacobsen KH, Pandit H, Lavy C. The burden of trauma in four rural district hospitals in Malawi: a retrospective review of medical records. Injury. 2014;45(12):2065–70. S0020-1383(14)00485-9 [pii]. 10.1016/j.injury.2014.10.001 25458068

[25] Jaffry Z, Chokotho LC, Harrison WJ, Mkandawire NC. The burden of trauma at a district hospital in Malawi. Tropical Doctor. 2017:49475517690333 10.1177/0049475517690333 28173743

[26] Chokotho L, Jacobsen KH, Burgess D, Labib M, Le G, Peter N, et al A review of existing trauma and musculoskeletal impairment (TMSI) care capacity in East, Central, and Southern Africa. Injury. 2016;47(9):1990–5. Epub 2016/05/15. 10.1016/j.injury.2015.10.036 . 27178767

[27] Malawi’s Health and Educational Systems2015 29 January 2019. Available from: https://seedglobalhealth.org/wp-content/uploads/2015/01/Malawis-Health-and-Educational-Systems.pdf.

[28] Mulwafu W, Chokotho L, Mkandawire N, Pandit H, Deckelbaum DL, Lavy C, et al Trauma care in Malawi: A call to action. Malawi Medical Journal. 2017;29(2):198–202. Epub 2017/09/29. 28955433 10.4314/mmj.v29i2.23 PMC5610296

[29] Lavy C, Tindall A, Steinlechner C, Mkandawire N, Chimangeni S. Surgery in Malawi—a national survey of activity in rural and urban hospitals. Annals of the Royal College of Surgeons of England. 2007;89(7):722–4. Epub 2007/10/26. 10.1308/003588407X209329 17959015 PMC2121267

[30] Chagomerana MB, Tomlinson J, Young S, Hosseinipour MC, Banza L, Lee CN. High morbidity and mortality after lower extremity injuries in Malawi: A prospective cohort study of 905 patients. International Journal of Surgery (London, England). 2017;39:23–9. S1743-9191(17)30050-X [pii]. 10.1016/j.ijsu.2017.01.047 28110030

[31] Agarwal-Harding KJ, Meara JG, Greenberg SL, Hagander LE, Zurakowski D, Dyer GS. Estimating the global incidence of femoral fracture from road traffic collisions: a literature review. The Journal of Bone and Joint Surgery American volume. 2015;97(6):e31 10.2106/JBJS.N.00314 25788312

[32] Agarwal-Harding KJ, Chokotho LC, Young S, Mkandawire N, Losina E, Katz JN. The prevalence and incidence of adults with femoral shaft fracture receiving care in Malawian district and central hospitals. Malawi Medical Journal. 2019; Submitted to Journal 11 May 2019.

[33] Lau BC, Wu HH, Mustafa M, Ibrahim J, Conway D, Agarwal-Harding K, et al Developing Research to Change Policy: Design of a Multicenter Cost-Effectiveness Analysis Comparing Intramedullary Nailing to Skeletal Traction in Malawi. Journal of Orthopaedic Trauma. 2018;32 Suppl 7:S52–S7. Epub 2018/09/25. 10.1097/BOT.0000000000001299 . 30247402

[34] Stewart BT, Quansah R, Gyedu A, Ankomah J, Donkor P, Mock C. Strategic Assessment of Trauma Care Capacity in Ghana. World Journal of Surgery. 2015;39(10):2428–40. 10.1007/s00268-015-3132-3 26154575

[35] Mock C, Lormand JD, Goosen J, Joshipura M, Peden M. Guidelines for essential trauma care. Geneva: World Health Organization; 2004.

[36] Alarcon MDL, Estevez FV, Cabezon-Gutierrez L, Padros MC, Martin-Arroyo JMT, Rebollo MA, et al Expert consensus on the management of breakthrough cancer pain in older patients. A Delphi study. Journal of Geriatric Oncology. 2019 Epub 2019/05/01. 10.1016/j.jgo.2019.03.012 . 31036463

[37] Audige L, Flury M, Muller AM, Durchholz H. Complications associated with arthroscopic rotator cuff tear repair: definition of a core event set by Delphi consensus process. Journal of Shoulder and Elbow Surgery. 2016;25(12):1907–17. Epub 2016/08/09. 10.1016/j.jse.2016.04.036 . 27496354

[38] Yu A, Devine CA, Kasdin RG, Orizondo M, Perdomo W, Davis AM, et al Pain management among Dominican patients with advanced osteoarthritis: a qualitative study. BMC Musculoskelet Disorders. 2016;17:y. 10.1186/s12891-016-1075-y 27184397 PMC4869371

[39] McHugh ML. Interrater reliability: the kappa statistic. Biochemia Medica. 2012;22(3):276–82. 23092060 PMC3900052

[40] Grimes CE, Mkandawire NC, Billingsley ML, Ngulube C, Cobey JC. The cost-effectiveness of orthopaedic clinical officers in Malawi. Tropical Doctor. 2014;44(3):128–34. 10.1177/0049475514535575 [pii]. 24821618

[41] Mkandawire N, Ngulube C, Lavy C. Orthopaedic clinical officer program in Malawi: a model for providing orthopaedic care. Clinical Orthopaedics and Related Research. 2008;466(10):2385–91. 10.1007/s11999-008-0366-5 18633684 PMC2584281

[42] Cornelissen D, Mwapasa G, Gajewski J, McCauley T, Borgstein E, Brugha R, et al The Cost of Providing District-Level Surgery in Malawi. World Journal of Surgery. 2018;42(1):46–53. Epub 2017/08/10. 10.1007/s00268-017-4166-5 28791448 PMC5740194

[43] Foundation Stone Laying Ceremony for the Lilongwe Institute of Orthopaedics and Neurosurgery (LION): AO Alliance; 2018 [updated November 14, 2018; cited 2019 January 15]. Available from: https://ao-alliance.org/news/foundation-stone-laying-ceremony-for-the-lilongwe-institute-of-orthopaedics-and-neurosurgery-lion/.

[44] Yoon RS, Liporace FA. Impact of Intramedullary Nailing in the Treatment of Femur Fractures An Evolutionary Perspective. Bulletin of the NYU Hospital for Joint Diseases. 2018;76(1):9–13. Epub 2018/03/15. . 29537951

[45] Eaton J, Grudziak J, Hanif AB, Chisenga WC, Hadar E, Charles A. The effect of anatomic location of injury on mortality risk in a resource-poor setting. Injury. 2017;48(7):1432–8. S0020-1383(17)30348-0 [pii]. 10.1016/j.injury.2017.05.023 28551054

[46] Agarwal-Harding KJ, Chokotho LC, Mkandawire NC, Martin C Jr., Losina E, Katz JN. Risk Factors for Delayed Presentation Among Patients with Musculoskeletal Injuries in Malawi. The Journal of Bone and Joint Surgery American volume. 2019;101(10):920–31. Epub 2019/05/17. 10.2106/JBJS.18.00516 . 31094984 PMC6530973

