# Supplementary material for: Assessing the capacity of Malawi’s district and central hospitals to manage traumatic diaphyseal femoral fractures in adults
Source: PLoS One. 2019 Nov 20;14(11):e0225254. doi: 10.1371/journal.pone.0225254 (PMC6867700; doi:10.1371/journal.pone.0225254)
Supplement: S1 File — Includes additional study context regarding musculoskeletal trauma care and femoral shaft fractures in Malawi, and the capacity assessment survey tool. (DOCX) [file pone.0225254.s001.docx]

Assessing the capacity of Malawi’s district and central hospitals to manage traumatic diaphyseal femoral fractures in adults

**Supplementary Appendix**

Study Context 2

Musculoskeletal trauma care in Malawi 2

Femoral shaft fracture in Malawi 4

Capacity assessment survey tool 6

References 18

# Study Context

## **Musculoskeletal trauma care in Malawi**

Taking into consideration surgical capacity, safety, timeliness, and affordability of care, quality surgery remains inaccessible for 5 billion people worldwide, including 85% of people living in LMICs [1]. In many LMICs, orthopedic surgical care is deficient in infrastructure, manpower, and essential resources at all tiers but most significantly at the rural or district hospital level [2-7].

Malawi is a low-income country located in southeastern sub-Saharan Africa, bordering Mozambique, Zambia, and Tanzania. It has a population of about 19 million people, 83% of whom live in rural areas [8]. Half of the population lives below the national poverty line [9]. GDP per capita is $338, and annual health expenditure per capita is $34 [10]. Malawi’s health sector is one of the most donor-dependent worldwide, with the majority of total health expenditures provided by foreign donors [11]. The majority of health care services are provided by the government. However, the Christian Health Association of Malawi (CHAM), the largest non-governmental healthcare provider, provides an estimated 37% of the total healthcare services in Malawi [12, 13]. The extent to which musculoskeletal trauma care is offered by mission hospitals in Malawi remains unknown.

Under a national health plan, all services delivered by the Ministry of Health are free at the point of delivery in all government hospitals [14]. However, indirect costs of seeking care, especially transportation costs to reach a health facility, can still make healthcare inaccessible to many Malawians [14, 15]. There is no organized emergency response system in Malawi, and very few patients are brought to hospital by emergency medical services or by ambulance. Most patients reach hospital by private vehicle, public transportation, bicycle, or by foot [16, 17]. One-quarter of deaths in Malawi could be avoided with better access to surgical care, and one-third of Malawians in the community are living with an untreated surgical condition, 30% of which are of the upper or lower extremities [15].

Malawi has 41 practicing surgeons, including 11 orthopaedic specialists, yielding 0.24 surgeons per 100,000 people compared to 29 surgeons per 100,000 people in HIC [18, 19]. Fewer than half of all surgeons practice outside a major commercial city in Malawi [18]. To address this shortage in manpower, especially in the rural areas, Malawi has for the last 3 decades invested in the training and deployment of orthopaedic clinical officers (OCOs), or non-physician clinicians, who now manage up to 90% of orthopaedic workload. They are deployed primarily in rural areas and specialize primarily in conservative management of common traumatic injuries [20, 21]. In a survey of 27 government hospitals in Malawi, over 90% of the surgical workforce were non-surgeons. Non-surgeons were the only providers of surgical services in over 85% of hospitals surveyed in Malawi, with no specialists serving the rural district hospitals [22].

However, despite efforts to boost manpower, significant limitations in essential resources and infrastructure exist at district and central hospitals in Malawi. In a survey of 267 district and referral hospitals throughout East, Central, and Southern Africa (including Malawi), only 31% had formal emergency departments to manage traumatic injuries. Mobile c-arm and CT scanning was available in fewer than 5% of district hospitals and in only 25% of central hospitals. While closed fracture care was available in 72% of hospitals, the tools necessary to perform surgical treatment of fractures were available in only 20% of district and 49% of central hospitals, with only 4% of district and 10% of central hospitals reporting a sustainable supply of implants [23].

The public hospital system in Malawi has three tiers: rural health centers, providing primary care; district hospitals, staffed by OCOs; and central (regional referral) hospitals in urban centers staffed by OCOs and orthopaedic surgeons. Health centers do not have orthopaedic staff, x-ray equipment to diagnose musculoskeletal injuries or basic equipment for temporary stabilization or definitive treatment of fractures. Patients presenting to health centers with fractures are therefore typically referred to a district or a central hospital for treatment. In Malawi, musculoskeletal trauma care, therefore, is delivered at 25 rural district hospitals and 4 urban central hospitals (S1 Table).

## **Femoral shaft fracture in Malawi**

Femoral shaft fractures alone, with an estimated worldwide incidence as high as 2.9 million annually, may outnumber new HIV infections worldwide, with rates in LMICs double those in high-income countries (HICs) [24, 25]. An estimated 2,362 femoral shaft fractures (estimated range: 1,212 to 3,511) occur annually in Malawi, with an incidence rate of 22.6 per 100,000 [24, 26]. We have previously reported our findings from a nationwide cross-sectional survey of adult patients with femoral shaft fracture seen in Malawian district and central hospitals. These data were collected in conjunction with the capacity assessment, by survey provided to OCOs at each hospital between May 29^th^ and June 15^th^, 2018. At the time of the survey, 120 patients with femoral shaft fracture were currently receiving treatment in a Malawian district or central hospital, with a median of three patients (IQR 2-5) at each district hospital and 10 (IQR 8-11) at each central hospital. Approximately one new patient (IQR 0-2) is admitted to each district hospital, and 4 (IQR 3-4) are admitted to each central hospital each week in Malawi [26]. The number of adult patients treated in government hospitals also appears to be increasing: a retrospective study at Kamuzu Central Hospital showed that 992 patients with femoral fractures were treated from 2009 to 2014, with an increase of 132% annually [27].

Adult patients with femoral shaft fractures are treated non-operatively with skeletal traction in the district hospital settings, and only treated operatively in the central hospitals [28]. The outcomes of patients with femoral shaft fracture treated in Malawi remains unknown.

# Capacity assessment survey tool

| **Capacity to Manage Adult Femoral Shaft Fracture**  **Confidential Research Survey** | |
| --- | --- |
| **Hospital: ___________________________________**  **Date: ______________________________________** | |
| How many adults with femoral shaft fractures were admitted to your hospital in the last 7 days?  How many adults with femoral shaft fractures are currently admitted to your hospital? | Please specify: _________  Please specify: _________ |
| ***Infrastructure*** | |
| Does your hospital have the capability of performing the following **labs** for patients:  Basic chemistry panel/ urea and electrolytes (U&E)?  *[Sodium, Potassium, Chloride, Bicarbonate, BUN, creatinine, glucose]*  Full blood count?  *(If yes to either question above)*  In the last 7 days, have labs ever been **unavailable** to those who need them?  *(If yes)*  Why? Please select one or more of the following: | Yes/No  Yes/No  Yes/No  a) Machinery broken and awaiting repairs  b) No staff member available at night or on the weekend  c) No staff member trained in using the available item  d) Necessary equipment, reagents, or other supplies out of stock or insufficient in number  e) No electricity to power machines  f) Other, please specify: ____________ |
| Does your hospital have a **casualty area/accident and emergency department**?  *(If yes)*  In the last 7 days, do you feel that the department was **unable** to effectively triage, diagnose, and stabilize adult patients with femur fracture?  *(If yes)*  Why? Please select one or more of the following: | Yes/No  Yes/No  a) Too few staff members  b) Staff members unavailable at night or on the weekend  c) Staff members inadequately trained  d) Necessary equipment or other supplies out of stock or insufficient in number  e) Other, please specify: ____________ |
| Does your hospital have at least one **ambulance** for same or next day transfer to another hospital?  *(If yes)*  Please state the number of ambulances at your hospital:  In the last 7 days, was an ambulance ever **unavailable** to transfer patients?  *(If yes)*  Why? Please select one or more of the following: | Yes/No  Number of ambulances: ______  Yes/No  a) Ambulance broken and awaiting repairs  b) No staff member available at night or on the weekend to drive ambulance  c) No staff member trained in driving the ambulance  d) Necessary equipment or other supplies out of stock or insufficient in number  e) No fuel for the ambulance  f) Ambulance in use elsewhere  g) Other, please specify: ____________ |
| Does your hospital have **inpatient hospital beds**?  *(If yes)*  In the last 7 days, have inpatient hospital beds ever been **unavailable** to admit patients?  *(If yes)*  Why? Please select one or more of the following: | Yes/No  Yes/No  a) Hospital beds broken, dirty, or otherwise unusable  b) More patients than beds available  c) Other, please specify: _______________ |
| Is there a **procedure room** to perform basic procedures (i.e. closed reduction, placement of skeletal traction pins)?  *(If yes)*  In the last 7 days, has the procedure room ever been **unavailable** to those who need to use it?  *(If yes)*  Why? Please select one or more of the following: | Yes/No  Yes/No  a) Too few staff members  b) Staff members unavailable at night or on the weekend  c) Staff members are inadequately trained  d) Necessary equipment or other supplies out of stock or insufficient in number  e) Room dirty or otherwise unusable  f) No electricity  g) No running water  h) Other, please specify: ____________ |
| Is there a functioning **operating theatre** available to perform manipulations under anesthesia and/or open reduction and internal fixation of fractures?  *(If yes)*  In the last 7 days, has the operating room ever been **unavailable** to those who need to use it?  *(If yes)*  Why? Please select one or more of the following: | Yes/No  Yes/No  a) Too few staff members  b) Staff members unavailable at night or on the weekend  c) Staff members are inadequately trained  d) Necessary equipment or other supplies out of stock or insufficient in number  e) Room dirty or otherwise unusable  f) No electricity  g) No running water  h) Other, please specify: ____________ |
| *(If operating room: yes)*  Is there a **post-anesthesia recovery room** (or equivalent) available for patients to recover after surgery?  If no, are nurses trained in post-anesthesia care available to care for patients after surgery?  *(If yes to either question above)*  In the last 7 days, did any adult patients with femur fracture receive **inadequate** post-anesthesia care?  *(If yes)*  Why? Please select one or more of the following: | Yes/No  Yes/No  Yes/No  a) Too few staff members  b) Staff members unavailable at night or on the weekend  c) Staff members inadequately trained  d) Necessary equipment or other supplies out of stock or insufficient in number  e) Other, please specify: ____________ |
| Is there a space dedicated to **rehabilitation and physiotherapy** for adult patients with treated femur fractures?  *(If yes)*  In the last 7 days, do you feel that any adult patients with femur fracture received **inadequate** physiotherapy and/or rehabilitation?  *(If yes)*  Why? Please select one or more of the following: | Yes/No  Yes/No  a) Too few staff members  b) Staff members unavailable at night or on the weekend  c) Staff members inadequately trained  d) Necessary equipment or other supplies out of stock or insufficient in number  e) Other, please specify: ____________ |
| ***Manpower*** | |
| Is there a **triage nurse or equivalent** in the casualty/A&E department to assist in triage of patients with musculoskeletal trauma?  *(If yes)*  In the last 7 days, were they ever **unavailable** when needed? | Yes/No  Yes/No |
| Are there **clinical officers or doctors** in the casualty/A&E department to assist with initial work-up, diagnosis, and stabilization of adult patients with femur fracture?  *(If yes)*  In the last 7 days, were they ever **unavailable** when needed? | Yes/No  Yes/No |
| Is there at least one **radiology technician** in your hospital to perform x-rays and/or other imaging?  *(If yes)*  In the last 7 days, were they ever **unavailable** when needed? | Yes/No  Yes/No |
| Is there at least one **orthopaedic clinical officer (OCO)** at your hospital?  *(If yes)*  In the last 7 days, were they ever **unavailable** when needed? | Yes/No  Yes/No |
| Is there at least one **orthopaedic surgeon** at your hospital?  *(If yes)*  In the last 7 days, were they ever **unavailable** when needed? | Yes/No  Yes/No |
| Is there a **scrub nurse** to assist with procedures in the operating theater?  *(If yes)*  In the last 7 days, were they ever **unavailable** when needed? | Yes/No  Yes/No |
| Is there a **circulating nurse** to assist with procedures in the operative theater?  *(If yes)*  In the last 7 days, were they ever **unavailable** when needed? | Yes/No  Yes/No |
| Are there **ward nurses** to help care for admitted patients on the ward?  *(If yes)*  In the last 7 days, were there ever **too few nurses** to manage all patients? | Yes/No  Yes/No |
| Is there at least one **anesthesia clinical officer (ACO)** at your hospital?  *(If yes)*  In the last 7 days, were they ever **unavailable** when needed? | Yes/No  Yes/No |
| Is there an **anesthesiologist** at your hospital?  *(If yes)*  In the last 7 days, were they ever **unavailable** when needed? | Yes/No  Yes/No |
| Do you have staff available to assist with **discharge planning** (i.e. assisting disabled patients in accessing and utilizing community services after discharge)?  *(If yes)*  In the last 7 days, were they ever **unavailable** when needed? | Yes/No  Yes/No |
| Is there at least one trained **physiotherapist** at your hospital?  *(If yes)*  In the last 7 days, were they ever **unavailable** when needed? | Yes/No  Yes/No |
| Are there any **nurses trained in basic physiotherapy and/or rehabilitation** relevant to adult patients with femur fracture?  *(If yes)*  In the last 7 days, were they ever **unavailable** when needed? | Yes/No  Yes/No |
| ***Material Resources*** | |
| Is there a **pulse oximeter** in the hospital?  *(If yes)*  In the last 7 days, was it ever **unavailable** when needed?  *(If yes)*  Why? Please select one or more of the following: | Yes/No  Yes/No  a) Item broken and awaiting repairs  b) Item could not be located  c) Item in use elsewhere  d) Staff members are inadequately trained to use item  e) Other, please specify: _________________ |
| Is there a machine to perform an **electrocardiogram** (EKG)?  *(If yes)*  In the last 7 days, was it ever **unavailable** when needed?  *(If yes)*  Why? Please select one or more of the following: | Yes/No  Yes/No  a) Item broken and awaiting repairs  b) Item could not be located  c) Item in use elsewhere  d) Staff members unavailable at night or on the weekend to use item  e) Staff members are inadequately trained to use item  f) Necessary equipment or other supplies out of stock or insufficient in number  g) Other, please specify: ____________ |
| Is there a **thermometer**?  *(If yes)*  In the last 7 days, was it ever **unavailable** when needed?  *(If yes)*  Why? Please select one or more of the following: | Yes/No  Yes/No  a) Item broken and awaiting repairs  b) Item could not be located  c) Item in use elsewhere  d) Staff members are inadequately trained to use item  e) Other, please specify: _________________ |
| Is there a **blood pressure cuff**?  *(If yes)*  In the last 7 days, was it ever **unavailable** when needed?  *(If yes)*  Why? Please select one or more of the following: | Yes/No  Yes/No  a) Item broken and awaiting repairs  b) Item could not be located  c) Item in use elsewhere  d) Staff members are inadequately trained to use item  e) Other, please specify: _________________ |
| Are there available staff and a machine to perform **x-rays**?  *(If yes)*  Do you consistently get images of **adequate quality** to safely treat adult femur fractures?  In the last 7 days, was it ever **unavailable** when needed?  *(If yes)*  Why? Please select one or more of the following: | Yes/No  Yes/No  Yes/No  a) Item broken and awaiting repairs  b) Item could not be located  c) Staff members unavailable at night or on the weekend to use item  d) Staff members are inadequately trained to use item  e) Necessary equipment or other supplies out of stock or insufficient in number  f) Other, please specify: ____________ |
| Is there an **x-ray image intensifier (portable C-arm)** for use during procedures?  *(If yes)*  Do you consistently get images of **adequate quality** to safely treat adult femur fractures?  In the last 7 days, was it ever **unavailable** when needed?  *(If yes)*  Why? Please select one or more of the following: | Yes/No  Yes/No  Yes/No  a) Item broken and awaiting repairs  b) Item could not be located  c) Item in use elsewhere  d) Staff members unavailable at night or on the weekend to use item  e) Staff members are inadequately trained to use item  f) Necessary equipment or other supplies out of stock or insufficient in number  g) Other, please specify: ____________ |
| Is there a **CT scanner**?  *(If yes)*  Do you consistently get images with **adequate quality** to characterize complex fractures or complications (i.e. non-unions)?  In the last 7 days, was it ever **unavailable** when needed?  *(If yes)*  Why? Please select one or more of the following: | Yes/No  Yes/No  Yes/No  a) Item broken and awaiting repairs  b) Item could not be located  c) Staff members unavailable at night or on the weekend to use item  d) Staff members are inadequately trained to use item  e) Necessary equipment or other supplies out of stock or insufficient in number  f) Other, please specify: ____________ |
| Is there an **ultrasound machine**?  *(If yes)*  Do you consistently get images of **adequate quality** to diagnose deep vein thrombosis?  In the last 7 days, was it ever **unavailable** when needed?  *(If yes)*  Why? Please select one or more of the following: | Yes/No  Yes/No  Yes/No  a) Item broken and awaiting repairs  b) Item could not be located  c) Staff members unavailable at night or on the weekend to use item  d) Staff members are inadequately trained to use item  e) Necessary equipment or other supplies out of stock or insufficient in number  f) Other, please specify: ____________ |
| Does the normally hospital have the following **pain medications**:  morphine (or equivalent)?  Ibuprofen (or equivalent)?  Paracetamol/acetaminophen?  *(If yes to any of the above)*  In the last 7 days, was pain medication ever **unavailable** when needed? | Yes/No  Yes/No  Yes/No  Yes/No |
| Are there **intravenous infusion sets (lines and cannulas)**?  *(If yes)*  In the last 7 days, were they ever **unavailable** when needed? | Yes/No  Yes/No |
| Is there a supply of **crystalloids** (i.e. normal saline, lactated ringers) for fluid resuscitation?  *(If yes)*  In the last 7 days, were they ever **unavailable** when needed? | Yes/No  Yes/No |
| Is there a supply of **colloids** (i.e. albumin, fresh frozen plasma) for fluid resuscitation?  *(If yes)*  In the last 7 days, were they ever **unavailable** when needed? | Yes/No  Yes/No |
| Is there a supply of **blood products**?  *(If yes)*  In the last 7 days, were they ever **unavailable** when needed? | Yes/No  Yes/No |
| Is there a supply of **general anesthetic reagents** (i.e. ether, halothane, or equivalent)?  *(If yes)*  In the last 7 days, were they ever **unavailable** when needed? | Yes/No  Yes/No |
| Is there a supply of **oral antibiotics**?  Is there a supply of **IV antibiotics**?  *(If yes to either question)*  In the last 7 days, were they ever **unavailable** when needed? | Yes/No  Yes/No  Yes/No |
| Does the hospital normally have the following **blood-thinning medications**:  Acetylsalicylic acid (aspirin)?  Low molecular weight heparin (or equivalent)?  *(If yes to any of the above)*  In the last 7 days, were blood-thinning medications ever **unavailable** when needed? | Yes/No  Yes/No  Yes/No |
| Can you perform **skin traction** at your hospital?  *(If yes)*  In the last 7 days, were you **unable** to perform skin traction when needed?  *If you were ever unable to perform skin traction,*  Why? Please select one or more of the following: | Yes/No  Yes/No  a) Inadequate supply of tape  b) Inadequate supply of weights  c) Inappropriate or inadequate supply of hospital beds with traction frames  d) Other missing supplies: _________________  ______________________________________  e) Staff members unavailable at night or on the weekend  f) Staff members are inadequately trained to perform skin traction  g) Staff members are inadequately trained to care for patients in skin traction  h) Other, please specify: ____________ |
| Can you perform **skeletal traction** at your hospital?  *(If yes)*  In the last 7 days, were you **unable** to perform skeletal traction when needed?  *If you were ever unable to perform skeletal traction,*  Why? Please select one or more of the following: | Yes/No  Yes/No  a) Drill unavailable or absent  b) Inadequate supply of traction pins  c) Inadequate supply of weights  d) Inadequate supply of local anesthetic  e) Inappropriate or inadequate supply of hospital beds with traction frames  f) Other missing supplies: _________________  ______________________________________  g) Staff members unavailable at night or on the weekend  h) Staff members are inadequately trained to perform skeletal traction  i) Staff members are inadequately trained to care for patients in skeletal traction  j) Other, please specify: ____________ |
| Is there normally **Plaster of Paris** (PoP)?  In the last 7 days, were you ever/at any time **unable** to use PoP to splint or otherwise stabilize a fracture?  *If you were ever unable to use PoP,*  Why? Please select one or more of the following: | Yes/No  Yes/No  a) Inadequate supply  b) Item could not be located  c) Staff members unavailable at night or on the weekend to use item  d) Staff members are inadequately trained to use item to stabilize fractures (splint or cast)  e) No running water  f) Other, please specify: ____________ |
| Is there at least one **external fixator** in the hospital?  In the last 7 days, were you **unable** to use an external fixator to stabilize a fracture you felt needed this?  *(If yes)*  Why? Please select one or more of the following: | Yes/No  Yes/No  a) Item broken and awaiting repairs  b) Item could not be located  c) Staff members unavailable at night or on the weekend to use item  d) Staff members are inadequately trained to use item  e) Necessary equipment or other supplies out of stock or insufficient in number  f) Item(s) not sterile  f) Other, please specify: ____________ |
| Does your hospital have **intramedullary nails** for internal fixation of adult femur fractures?  Is there a **sustainable supply** of implants?  If yes, in the last 7 days, were you **unable** to use an intramedullary nail to treat a patient that would benefit from this?  *(If yes)*  Why? Please select one or more of the following: | Yes/No  Yes/No  Yes/No  a) Operating room unavailable  b) Item could not be located  c) Staff members unavailable at night or on the weekend to use item  d) Staff members are inadequately trained to use item  e) Necessary equipment or other supplies out of stock or insufficient in number  f) Other, please specify: ____________ |
| Does your hospital have **large fragment plates and screws** for internal fixation of adult femur fractures?  Is there a **sustainable supply** of implants?  In the last 7 days, were you **unable** to use plates and screws to internally fix a fracture?  *(If yes)*  Why? Please select one or more of the following: | Yes/No  Yes/No  Yes/No  a) Operating room unavailable  b) Item could not be located  c) Staff members unavailable at night or on the weekend to use item  d) Staff members are inadequately trained to use item  e) Necessary equipment or other supplies out of stock or insufficient in number  f) Other, please specify: ____________ |
| Is there a supply of **suture**?  *(If yes)*  In the last 7 days, was suture ever **unavailable** when needed? | Yes/No  Yes/No |
| Is there a supply of sterile **cotton wool** for use in the operating theatre?  *(If yes)*  In the last 7 days, was cotton wool ever **unavailable** when needed? | Yes/No  Yes/No |
| Is there a supply of **gauze and bandages**?  *(If yes)*  In the last 7 days, were they ever **unavailable** when needed? | Yes/No  Yes/No |
| Is there a supply of **walking assistive devices** (i.e. walkers/frames, canes, crutches)?  *(If yes)*  In the last 7 days, were they ever **unavailable** when needed? | Yes/No  Yes/No |

# References

1. Alkire BC, Raykar NP, Shrime MG, Weiser TG, Bickler SW, Rose JA, et al. Global access to surgical care: a modelling study. The Lancet Global Health. 2015;3(6):316. doi: 10.1016/S2214-109X(15)70115-4 [doi].

2. Spiegel DA, Nduaguba A, Cherian MN, Monono M, Kelley ET. Deficiencies in the availability of essential musculoskeletal surgical services at 883 health facilities in 24 low- and lower-middle-income countries. World Journal of Surgery. 2015;39(6):1421-32. doi: 10.1007/s00268-015-2971-2 [doi].

3. Shrime MG, Daniels KM, Meara JG. Half a billion surgical cases: Aligning surgical delivery with best-performing health systems. Surgery. 2015;158(1):27-32. doi: 10.1016/j.surg.2015.03.025 [doi].

4. The world health report 2006: working together for health. Geneva, Switzerland: World Health Organization; 2006.

5. Funk LM, Weiser TG, Berry WR, Lipsitz SR, Merry AF, Enright AC, et al. Global operating theatre distribution and pulse oximetry supply: an estimation from reported data. Lancet (London, England). 2010;376(9746):1055-61. doi: 10.1016/S0140-6736(10)60392-3 [doi].

6. Meara JG, Leather AJ, Hagander L, Alkire BC, Alonso N, Ameh EA, et al. Global Surgery 2030: evidence and solutions for achieving health, welfare, and economic development. Lancet (London, England). 2015;386(9993):569-624. doi: 10.1016/S0140-6736(15)60160-X [doi].

7. Linden AF, Sekidde FS, Galukande M, Knowlton LM, Chackungal S, McQueen KA. Challenges of surgery in developing countries: a survey of surgical and anesthesia capacity in Uganda's public hospitals. World Journal of Surgery. 2012;36(5):1056-65. doi: 10.1007/s00268-012-1482-7 [doi].

8. Health Nutrition and Population Statistics [Internet]. The World Bank. 2017 [cited March 20, 2019]. Available from: <https://datacatalog.worldbank.org/dataset/health-nutrition-and-population-statistics>.

9. Poverty and Equity Database [Internet]. The World Bank. 2016 [cited January 15, 2019]. Available from: <https://datacatalog.worldbank.org/dataset/poverty-and-equity-database>.

10. World Development Indicators [Internet]. The World Bank. 2017 [cited March 20, 2019]. Available from: <https://datacatalog.worldbank.org/dataset/world-development-indicators>.

11. USAID. Malawi Health System Strengthening Fact Sheet 2016 [updated September 15, 2016; cited 2019 January 15]. Available from: <https://www.usaid.gov/malawi/fact-sheets/malawi-health-systems-strengthening-fact-sheet>.

12. Malawi’s Health and Educational Systems2015 29 January 2019. Available from: <https://seedglobalhealth.org/wp-content/uploads/2015/01/Malawis-Health-and-Educational-Systems.pdf>.

13. Lavy C, Tindall A, Steinlechner C, Mkandawire N, Chimangeni S. Surgery in Malawi - a national survey of activity in rural and urban hospitals. Annals of the Royal College of Surgeons of England. 2007;89(7):722-4. Epub 2007/10/26. doi: 10.1308/003588407X209329. PubMed PMID: 17959015; PubMed Central PMCID: PMC2121267.

14. Second Generation, WHO Country Cooperation Strategy, 2008-2013, Malawi. WHO Regional Office for Africa. 2009.

15. Varela C, Young S, Groen R, Banza L, Mkandawire NC, Viste A. Untreated surgical conditions in Malawi: A randomised cross-sectional nationwide household survey. Malawi Medical Journal. 2017;29(3):231-6. Epub 2018/06/07. doi: 10.4314/mmj.v29i3.1. PubMed PMID: 29872512; PubMed Central PMCID: PMCPMC5811994.

16. Samuel JC, Akinkuotu A, Villaveces A, Charles AG, Lee CN, Hoffman IF, et al. Epidemiology of injuries at a tertiary care center in Malawi. World Journal of Surgery. 2009;33(9):1836-41. doi: 10.1007/s00268-009-0113-4 [doi].

17. Jaffry Z, Chokotho LC, Harrison WJ, Mkandawire NC. The burden of trauma at a district hospital in Malawi. Tropical Doctor. 2017:49475517690333. doi: 10.1177/0049475517690333.

10.1177/0049475517690333 [doi].

18. O'Flynn E, Andrew J, Hutch A, Kelly C, Jani P, Kakande I, et al. The Specialist Surgeon Workforce in East, Central and Southern Africa: A Situation Analysis. World Journal of Surgery. 2016. doi: 10.1007/s00268-016-3601-3 [doi].

19. Holmer H, Lantz A, Kunjumen T, Finlayson S, Hoyler M, Siyam A, et al. Global distribution of surgeons, anaesthesiologists, and obstetricians. The LancetGlobal health. 2015;3 Suppl 2:9. doi: 10.1016/S2214-109X(14)70349-3 [doi].

20. Grimes CE, Mkandawire NC, Billingsley ML, Ngulube C, Cobey JC. The cost-effectiveness of orthopaedic clinical officers in Malawi. Tropical Doctor. 2014;44(3):128-34. doi: 0049475514535575 [pii].

21. Mkandawire N, Ngulube C, Lavy C. Orthopaedic clinical officer program in Malawi: a model for providing orthopaedic care. Clinical Orthopaedics and Related Research. 2008;466(10):2385-91. doi: 10.1007/s11999-008-0366-5 [doi].

22. Henry JA, Frenkel E, Borgstein E, Mkandawire N, Goddia C. Surgical and anaesthetic capacity of hospitals in Malawi: key insights. Health policy and planning. 2015;30(8):985-94. doi: 10.1093/heapol/czu102 [doi].

23. Chokotho L, Jacobsen KH, Burgess D, Labib M, Le G, Peter N, et al. A review of existing trauma and musculoskeletal impairment (TMSI) care capacity in East, Central, and Southern Africa. Injury. 2016;47(9):1990-5. Epub 2016/05/15. doi: 10.1016/j.injury.2015.10.036. PubMed PMID: 27178767.

24. Agarwal-Harding KJ, Meara JG, Greenberg SL, Hagander LE, Zurakowski D, Dyer GS. Estimating the global incidence of femoral fracture from road traffic collisions: a literature review. The Journal of Bone and Joint Surgery American volume. 2015;97(6):e31. doi: 10.2106/JBJS.N.00314 [doi].

25. Murray CJ, Ortblad KF, Guinovart C, Lim SS, Wolock TM, Roberts DA, et al. Global, regional, and national incidence and mortality for HIV, tuberculosis, and malaria during 1990-2013: a systematic analysis for the Global Burden of Disease Study 2013. Lancet (London, England). 2014;384(9947):1005-70. doi: 10.1016/S0140-6736(14)60844-8 [doi].

26. Agarwal-Harding KJ, Chokotho LC, Young S, Mkandawire N, Losina E, Katz JN. The prevalence and incidence of adults with femoral shaft fracture receiving care in Malawian district and central hospitals. Malawi Medical Journal. 2019; Submitted to Journal 11 May 2019.

27. Young S, Banza L, Munthali BS, Manda KG, Gallaher J, Charles A. The impact of the increasing burden of trauma in Malawi on orthopedic trauma service priorities at Kamuzu Central Hospital. Acta Orthopaedica. 2016;87(6):632-6. doi: 10.1080/17453674.2016.1228413 [doi].

28. Lau BC, Wu HH, Mustafa M, Ibrahim J, Conway D, Agarwal-Harding K, et al. Developing Research to Change Policy: Design of a Multicenter Cost-Effectiveness Analysis Comparing Intramedullary Nailing to Skeletal Traction in Malawi. Journal of Orthopaedic Trauma. 2018;32 Suppl 7:S52-S7. Epub 2018/09/25. doi: 10.1097/BOT.0000000000001299. PubMed PMID: 30247402.
