# Supplementary material for: Assessing the capacity of Malawi’s district and central hospitals to manage traumatic diaphyseal femoral fractures in adults
Source: PLoS One. 2019 Nov 20;14(11):e0225254. doi: 10.1371/journal.pone.0225254 (PMC6867700; doi:10.1371/journal.pone.0225254)
Supplement: S1 Table — (DOCX) [file pone.0225254.s002.docx]

**Assessing the capacity of Malawi’s district and central hospitals to manage traumatic diaphyseal femoral fractures in adults**

**S1 Table:** List of government district and central hospitals in Malawi.

| **District Hospitals** | | **Central Hospitals** | |
| --- | --- | --- | --- |
| **Name** | **Region** | **Name** | **Region** |
| Balaka District Hospital | Southern | Kamuzu Central Hospital | Central |
| Bwaila District Hospital | Central | Mzuzu Central Hospital | Northern |
| Chikwawa District Hospital | Southern | Queen Elizabeth Central Hospital | Southern |
| Chiradzulu District Hospital | Southern | Zomba Central Hospital | Southern |
| Chitipa District Hospital | Northern |  | |
| Dedza District Hospital | Central |  |  |
| Dowa District Hospital | Central |  |  |
| Karonga District Hospital | Northern |  |  |
| Kasungu District Hospital | Central |  |  |
| Machinga District Hospital | Southern |  |  |
| Mangochi District Hospital | Southern |  |  |
| Mchinji District Hospital | Central |  |  |
| Mulanje District Hospital | Southern |  |  |
| Mwanza District Hospital | Southern |  |  |
| Mzimba District Hospital | Northern |  |  |
| Neno District Hospital | Southern |  |  |
| Nkhatabay District Hospital | Northern |  |  |
| Nkhotakota District Hospital | Central |  |  |
| Nsanje District Hospital | Southern |  |  |
| Ntcheu District Hospital | Central |  |  |
| Ntchisi District Hospital | Central |  |  |
| Phalombe District Hospital | Southern |  |  |
| Rumphi District Hospital | Northern |  |  |
| Salima District Hospital | Central |  |  |
| Thyolo District Hospital | Southern |  |  |
